# Magnitude, Distribution, and Estimated Level of Underreporting of Acute Gastroenteritis in Jamaica

**Published:** 2013-12

**Authors:** Stephanie M. Fletcher, Eva Lewis-Fuller, Hank Williams, Zahra Miller, Henroy P. Scarlett, Collin Cooper, Kelly-Ann Gordon-Johnson, Ivan Vickers, Karen Shaw, Iyanna Wellington, Jennifer Thame, Enrique Pérez, Lisa Indar

**Affiliations:** ^1^Faculty of Health, University of Technology, Sydney, Australia; ^2^Ministry of Health, Jamaica; ^3^University of the West Indies, Mona, Jamaica; ^4^National Public Health Laboratory (NPHL), Jamaica; ^5^North East Regional Health Authority, Jamaica; ^6^Pan American Health Organization (PAHO), Panama; ^7^Caribbean Epidemiology Centre (CAREC/PAHO/WHO), Trinidad and Tobago

**Keywords:** Acute gastroenteritis, Burden of Illness Study, Communicable diseases, Foodborne illness, Population survey, Surveillance systems, Jamaica

## Abstract

Jamaica is the third largest island in the Caribbean. The epidemiology of acute gastroenteritis (AGE) is important to Jamaica, particularly in the areas of health, tourism, and because of the potential impact on the local workforce and the economy. Data collected by the National Surveillance Unit on the prevalence of AGE transmitted by food are not accurate. To determine the true magnitude, risk factors, and the extent of underreporting of AGE in Jamaica, we conducted a cross-sectional, population-based retrospective survey during the periods of 21 February–7 March and 14-27 June 2009, corresponding to high- and low-AGE season respectively. Of the total 1,920 persons selected randomly by a multistage cluster-sampling process, 1,264 responded (response rate 65.8%). Trained interviewers administered a standardized, validated questionnaire during face-to-face interviews. The overall prevalence of self-reported AGE was 4.0% (95% CI 2.9-5.1) at a rate of 0.5 episodes/per person-year. The highest monthly prevalence of AGE (14.6%) was found among the 1-4 year(s) age-group and the lowest (2.1%) among the 25-44 years age-group. Of the 18 cases (36%) who sought medical care, 11% were hospitalized, 33% were treated with antibiotics, and 66.7% received oral rehydration fluids. Only 2 cases who sought medical care reportedly submitted stool specimens. The mean duration of diarrhoea was 3.1 days, which resulted in a mean loss of 4 productive days, with over half of the cases requiring someone to care for them. The burden of syndromic AGE for 2009 was extrapolated to be 122,711 cases, showing an underreporting factor of 58.9. For every laboratory-confirmed AGE case, it was estimated that 383 more cases were occurring in the population. This research confirms that the prevalence of AGE is underreported in Jamaica and not being adequately detected by the current surveillance system. The components of the integrated surveillance system for AGE in Jamaica, particularly the laboratory aspect, need to be strengthened.

## INTRODUCTION

Jamaica is the third largest island in the Caribbean and is the largest of the English-speaking Commonwealth Caribbean Islands (1). The mid-year population in 2008 was estimated to be 2,687,241, with an average 1:1 gender distribution (2). Major sectors of the Jamaican economy include agriculture, manufacturing, tourism, and financial services, with tourism and mining being the leading earners of foreign exchange. An estimated 1.3 million foreign tourists visit Jamaica every year. The management and delivery of health services is delegated to four decentralized regional health authorities, namely South East, North East, Western, and Southern (3), each consisting of three or four parishes.

It is well-documented that diarrhoeal diseases are an important cause of morbidity and mortality worldwide (4). In the Caribbean region, diarrhoeal disease burden is thought to be high but there is little scientific evidence to support this claim. Data on acute gastroenteritis (AGE) reported to the Caribbean Epidemiology Centre (CAREC) from its member countries show high and increasing prevalence rates over the last 10 years (5,6). In Jamaica, the epidemiology of AGE, particularly on the emerging microorganisms and their prevalence in the community, is poorly understood. One of the reasons for lack of a comprehensive knowledge on AGE in Jamaica is that many people with AGE do not seek formal healthcare and are, thus, not captured by the National Surveillance Unit (NSU). Hence, the true burden of this illness is unknown, which limits implementation of appropriate preventive measures. Communicable disease surveillance for AGE in Jamaica is carried out by a passive system of voluntary case reporting by healthcare providers and laboratories (7). Syndromic AGE reports are submitted on a weekly basis from 61 sentinel sites to the NSU and show significant numbers (44,919 and 36,192 in 2006 and 2007 respectively) of reported cases of AGE (1). The 3 major laboratories that process AGE specimens—NPHL, CRH, and UHWI—report laboratory-confirmed foodborne pathogens, such as *Salmonella, Shigella, Campylobacter, Escherichia coli, Staphylococcus aureus*, norovirus, rotavirus, and parasites, to the NSU on a monthly basis. In 2005 and 2006, approximately half of the AGE cases reported to CAREC were from Jamaica (5). However, the exact proportion of AGE that is food-related had not been investigated in Jamaica. It should be noted that many pathogens transmitted through food are also spread through water or from person to person, thus obscuring the role of foodborne transmission (8).

Surveillance data, as single indicator of illness burden, represent only the tip of the epidemiological iceberg and, hence, cannot sufficiently describe full disease burden. Laboratory surveillance data identify the aetiology of AGE. However, it requires clinical correlation in order for the information to be utilized effectively. Cases may or may not be routinely captured in national data due to variations in healthcare-seeking behaviours, specimen collection, transportation, testing procedures, and reporting methods (5,9). The lack of understanding of the burden of AGE and extension of foodborne diseases (FBD) has undermined public health disease control efforts and impacted the allocation of resources. A lack of active research into the determinants of the magnitude and burden of common syndromic presentations, including AGE, has limited the opportunities for appropriate evidence-based action, prevention measures, and policy development. Accurate estimation of AGE burden is essential for managing the challenges relating to diarrhoeal illness and setting priorities for their prevention and control (10). Based on these challenges, there is a need to ascertain the baseline point prevalence of AGE to assess the true magnitude of the problem and provide information for strengthening the communicable disease surveillance systems in the future.

Our objective was to conduct a Burden of Illness (BOI) study to determine the magnitude, age distribution, and estimated level of underreporting of AGE to assess a more accurate burden of AGE in Jamaica. The data generated will provide a more complete understanding of the burden of AGE, its epidemiology, and the socioeconomic impact of this illness. This is necessary for mobilizing resources and initiating an effective response to prevention and control measures to reduce associated morbidity and mortality.

## MATERIALS AND METHODS

To assess the burden of AGE in Jamaica, the Ministry of Health (MOH) and CAREC used a cross-sectional survey design comprising population-based and laboratory-based surveys.

### Population-based study

Jamaica is divided into 14 parishes which are further subdivided into more than 5,000 Enumeration Districts (EDs), specifically-defined geographical areas, each consisting of up to 400 dwellings. Small contiguous EDs (having less than 100 dwellings) are amalgamated to form the primary sampling unit (PSU) for national surveys (11). The population-based study was performed with the administration of a standardized questionnaire to collect data on self-reported cases of AGE. The questionnaire was administered by trained healthcare workers in four regions. In addition to sociodemographic factors, the study sought information on the severity and symptoms associated with AGE experienced by respondents in the 30 days prior to the interview. The population survey was conducted over two periods (21 February–7 March 2009 and 14-27 June 2009), each covering 2 weeks that were selected based on the high- and low-prevalence seasons of AGE as determined by trends over the previous 5-year period (2003-2007). Acute gastroenteritis was defined as an episode of at least three loose stools within a 24-hour period, with or without associated symptoms.

### Sampling design

The population survey utilized a multistage cluster-sampling process, in which enumeration districts (EDs) or primary sampling units (PSUs) were randomly selected in each of the 14 parishes across Jamaica, followed by random selection of households and identification of the respondents/subjects within each household (11). The person selected to be interviewed was the individual who had most recently celebrated a birthday. If the eligible person was not at home at the time of visit, an appointment was made for a convenient time to revisit. After visiting a household twice without a response, that household was dropped from the sample. Adults (mother, father, or primary caregiver) were asked to answer for eligible children less than 18 years of age. However, another adult could not serve as a proxy for the other adult. Persons belonging to sub-populations considered to be vulnerable (prisoners and mentally-disabled persons) were excluded, in addition to infants below one year of age, persons who were not physically present in the country at the time of survey, and selected persons who were not at home for more than one month prior to the time of the survey.

### Sample-size

The sample-size was calculated using the Epi Info software (version 3.5.0) (12). A sample-size of 1,024 was calculated using the 2008 population of 2,687,241, based on an estimated prevalence of AGE of 40%, allowable error of 3%, and a 95% confidence interval. Based upon an expected response rate of 80% and a design effect of 1.5, the adjusted sample-size was 1,920. Of the total 1,920 individuals contacted to participate in the survey, there were 1,264 respondents (overall response rate 65.8%); 731 persons participated in Phase 1 (21 February–7 March 2009), and 533 persons participated in Phase 2 (14-27 June 2009).

### Laboratory survey

Laboratory diagnostic services are provided by the National Public Health Laboratory (NPHL) and laboratories of Cornwall Regional Hospital (CRH) and the University Hospital of the West Indies (UHWI). The smaller regional laboratories perform basic tests and refer samples to NPHL for confirmation and further testing. The NPHL is the country's only reference laboratory (1).

Laboratory surveys to examine records and data kept on AGE, including foodborne pathogens and laboratory practices, were conducted with the NPHL, UHWI and CRH laboratories during March 2009–February 2010. These facilities handle 80% of the AGE specimens processed in Jamaica and have an established reporting relationship with the National Surveillance System. Standard methods were used for isolating the foodborne pathogens, including *Salmonella, Shigella, Campylobacter, Staphylococci*, pathogenic *Escherichia coli,* norovirus, rotavirus, and enteric parasites (13). Monthly reports were reviewed to assess the number of samples submitted for AGE, test procedures, and the proportion that tested positive for pathogens, including *Salmonella, Shigella, Campylobacter, Staphylococci,* pathogenic *Escherichia coli*, norovirus, rotavirus, and enteric parasites.

### Ethical approval

Ethical approval for the study was granted by the MOH Advisory Panel on Ethics and Medico-legal Affairs and the Ethical Review Board of the UHWI. Written informed consent and/or assent for eligible individuals were obtained either directly from study participants (adults) or the parent or primary caregiver of eligible children aged less than 18 years. Confidentiality of respondents’ information was maintained by utilizing unique identification numbers, instead of personally identifying information on the questionnaire.

### Data analysis

Data were entered in duplicate using Epi Info (version 6.0) by trained data-entry clerks. Data were then exported into SPSS (version 13.0) and Intercooled STATA (version 9.0) for analysis. Univariate analysis was conducted to determine the monthly prevalence of AGE by sociodemographic variable to ascertain the frequencies and assess any significant association between the variables and the primary outcome of being a case of AGE. Persons who either did not indicate the number of stools or indicated that they had less than 3 loose stools within a 24-hour period were excluded from the analysis.

### Estimation of underreporting and burden of AGE

**Figure 1.**
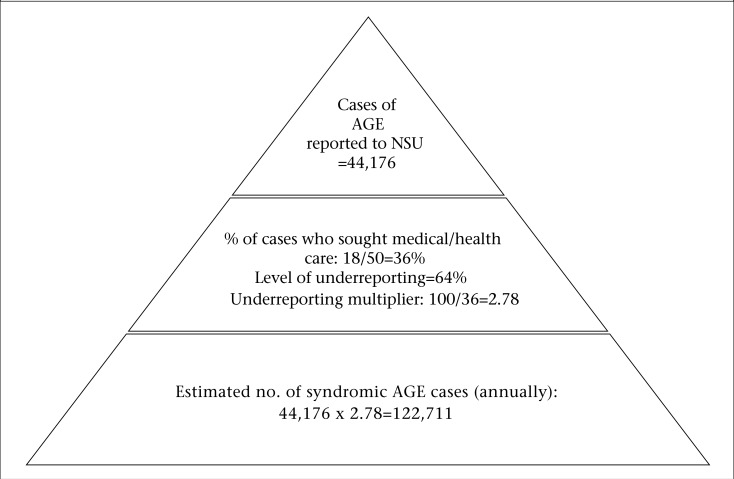
Estimation of underreporting for syndromic AGE in Jamaica, 2009

**Figure 2.**
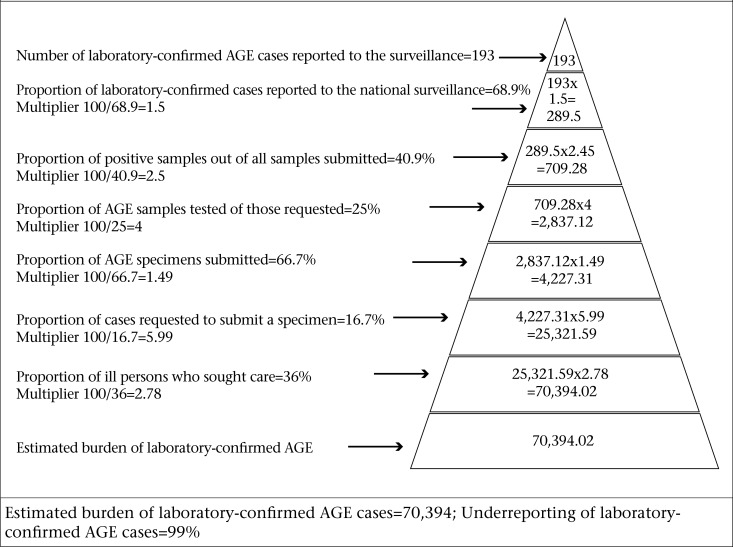
Estimation of underreporting for laboratory-confirmed AGE in Jamaica, 2009

The number of syndromic and laboratory-confirmed AGE cases reported to the National Surveillance System was adjusted with the data collected from the population and laboratory surveys to produce national estimates of the full burden of AGE and the extent of underreporting, using Burden of Illness pyramids as presented in Figure 1-2.

## RESULTS

### Representativeness of study population and response rate

Of the total 1,920 individuals contacted to participate in the survey, 1,264 responded (overall response rate 65.8%); 731 persons participated in Phase 1 (21 February–7 March 2009), and 533 persons participated in Phase 2 (14-27 June 2009). Table 1 illustrates the distribution of respondents based on their gender, age-group, and other sociodemographic variables. The gender distribution showed that 39.6% were male, and 60.4% were female; the sample consisted primarily of persons of African/Black descent (95.4%). The age-group with the highest representation was the 25-44 years (29.7%), followed by the 5-14 years (22.8%) and the 15-24 years (18.6%). The occupation most frequently reported was ‘service workers/shop-market sales persons’ (n=172,13.6%), followed by ‘professionals, ‘senior officials’ and ‘technicians’ (n=131,10.4%), and skilled agriculture/fishery workers (n=104, 8.2%). There were, however, 27.7% who did not specify their occupations and 23.7% who had no previous occupations. The most frequently-reported educational level attained by mothers was ‘secondary’ (605 or 47.9%), followed by ‘primary’ (n=307) and tertiary level, i.e. college and university degrees (n=177). Fathers, being underrepresented in the study compared to the general population, tended to have lower levels of education compared to mothers. However, nearly three times higher number of men (n=159) failed to indicate their level of education compared to women (46).

### Magnitude of illness

The monthly prevalence of AGE was estimated to be 4.0% (95% CI 2.9-5.1), resulting in an annual incidence of 0.5 episodes per person-year for the general population. The annual incidence per person-year was higher for males (0.7) and lower for females (0.4). Similar monthly prevalence was noted among both the genders. The highest monthly prevalence (14.6%) was among the 1-4 year(s) age-group and the lowest among the 25-44 years age-group (2.1%) (Table 1 and 2). Univariate and multinominal logistic regression analyses were conducted on the sociodemographic variables to test for significant relationship at p<0.05 but only the age-group of respondents turned out to be significantly associated with cases of acute gastroenteritis (p=0.001), particularly the 1-4 year(s) age-group. A higher proportion (75.7%) of self-reported AGE cases was reported on rural communities compared to urban communities (24.3%). The 1-4 year(s) age-group generally continued to show the highest prevalence of the disease in both rural and urban settings. However, due to the low level of responses, a monthly prevalence could not be calculated for several variables, and no significant differences were found even when the prevalence rates were weighted to represent the entire population and compared with the prevalence data in this study.

### Severity and associated symptoms

The associated symptoms and burden of illness among self-reported AGE cases are outlined in Table 3 and 4. The most common symptoms associated with AGE were abdominal pain (63.6%), sneezing (38.6%), cough (37.2%), vomiting (34.9%), and headache (34.1%). Only two cases reported having had blood in the stools. Fever (23.5%) did not emerge as a significant symptom associated with AGE in this study. The mean duration of AGE symptoms was 3.1 days [range 1-14 day(s)], which resulted in a mean loss of approximately 4 productive days [range 1-21 day(s)] from routine activities. The proportion of cases whose usual activities were restricted during their AGE-related illness was 76% or 38 out of 50 cases. In addition, approximately half (24/50) of the AGE cases required a caregiver during their illness for an average of 4.7 days.

### Healthcare-seeking behaviour/Use of medical systems

Only 36% (18/50) of self-reported cases of AGE indicated that they sought healthcare for their symptoms (Table 5). Of those who sought healthcare, 55.6% visited a public hospital, followed by seeking care at the private doctors’ clinics (27.8%) and health centres (16.7%); 11% required hospitalization, for whom the mean duration of hospitalization was 3.5 nights. One patient visited a private hospital, and another visited a traditional healer. All persons who reported seeking medical care had treatment prescribed, of which oral rehydration salts solution (ORS) was the most frequent remedy (66.7%). Pain killers and antibiotics were each prescribed to 33.3% of the cases. Non-prescribed treatments, including herbal and home remedies, were taken by 34% of the cases.

### Laboratory survey and practices

Stool specimens were requested from only 3 of the 18 cases (16.7%), and 2 of the 3 (66.7%) persons submitted specimens to the laboratory. Hence, majority of the AGE cases (16/18 or 88.9%) were not laboratory-tested to determine the aetiology of the AGE. Preliminary results of tests that have public-health significance, such as the aetiologies of AGE and suspected foodborne disease outbreaks, are reported from the NPHL to the National Surveillance Unit in the Ministry of Health immediately and followed up with definitive results later. The two cases who submitted stools for testing would have been reported from two sources to the surveillance system—the laboratory and the healthcare practitioner. During a period of laboratory surveillance in parallel with the study (March 2009 to February 2010), 280 stool specimens from persons with AGE were submitted but only 193 (68.9%) were reported to the NSU as having tested positive.

**Table 1.** Sociodemographic characteristics of respondents and estimated prevalence of acute gastroenteritis (AGE) in Jamaican communities in 2009

Variable	Residents (N)	Respondents (n)	Number ill	Monthly prevalence (%)	95% CI
Sex (p=0.88)					
Male		469	20	4.3	2.5-6.2
Female		715	29	4.1	2.7-5.6
Overall		1,242	50	4.0	
Age (completed years) (p=0.00)					
1-4	225,396*	90	13	14.6	7.2-22.0
5-14	529,031	209	10	4.8	1.9-7.6
15-24	457,722	212	8	3.8	1.2-6.4
25-44	850,479	341	7	2.1	0.6-3.6
45-64	399,065	242	8	3.4	1.1-5.8
≥65	225,549	159	4	2.5	0.1-5.0
Cultural group (p=0.80)					
African/Black		1,149		4.2	3.1-5.4
Other		56		-	-
Urban/rural distribution (0.30)					
Urban		618	21	3.4	2.0-4.9
Rural		646	29	4.6	3.0-6.2
Educational level of mother (n=1,180; p=0.77)					
Primary		307	8	2.7	0.8-4.5
Secondary		605	29	4.9	3.1-6.9
College		97	4	-	-
Undergraduate/Graduate		49	2	-	-
Postgraduate		31	1	-	-
Other		45	1	-	-
Don't know		46	1	-	-
Educational level of father (n=1,060; p=0.30)					
Primary		278	5	-	
Secondary		484	26	5.5	3.4-7.5
College		47	2	-	-
Undergraduate/Graduate		35	1	-	-
Postgraduate		25	1	-	-
Other		32	0	-	-
Don't know		159	7	-	-

*Population data for age-group 1-4 year(s) include persons aged <1 year

**Table 2.** Summary of prevalence and incidence of acute gastroenteritis (AGE) in Jamaica in 2009

Incidence/Prevalence	%
Annual incidence per person-year in general	0.5
Annual incidence per person-year in males	0.7
Annual incidence per person-year in females	0.4
Prevalence by age-group distribution of cases (completed years)	
1-4	14.6
5-14	4.8
15-24	3.8
25-44	2.1
45-64	3.4
≥65	2.5

**Table 3.** Symptoms associated with self-reported cases of acute gastroenteritis (AGE) in Jamaica, 2009

Symptom	Number of cases (%)
Fever (measured) (n=31)	5 (16.1)
Fever (not measured) (n=34)	8 (23.5)
Blood in stool (n=40)	2 (5)
Vomiting (n=43)	15 (34.9)
Abdominal pain (n=44)	28 (63.6)
Headache (n=44)	15 (34.1)
Nausea (n=40)	9 (22.5)
Sore throat (n=41)	6 (14.6)
Cough (n=43)	16 (37.2)
Runny nose (n=41)	11 (26.8)
Sneezing (n=44)	17 (38.6)
Other (n=40)	2 (5)

**Table 4.** Duration of illness, productive days lost, and caregiver's days required among self-reported cases of acute gastroenteritis (AGE) in Jamaica, 2009

Variable	Mean	Median	Range
Duration of illness (days) (n=44)	3.1	2	1-14
Productive days lost (n=38)	4.1	2	1-21
Caregiver's days (n=22)	4.7	3	1-21

### Estimation of underreporting and overall burden of AGE

The total number of AGE cases reported to NSU during one year period (March 2009–February 2010) was 44,176. From the population survey, it was estimated that, for every case reported to NSU, there were 2.78 additional cases in the community. Using this multiplier, it was estimated that the burden of illness for syndromic AGE in Jamaica for the period March 2009–February 2010 was 122,711 cases. This indicates a 64.0% underreporting to the surveillance system (Figure 1). Furthermore, the laboratory-based AGE-related burden of illness was 70,394 (Figure 2), with an underreporting factor of 364.74, indicating a high level of underreporting of laboratory-tested cases. Thus, for every laboratory-confirmed AGE case, there were 365 unreported cases occurring in the community.

## DISCUSSION

This was the first study to provide a population-based estimate of the self-reported prevalence and underreporting of AGE in Jamaica and the risk factors associated with AGE and foodborne diseases (FBD), which provided valuable and necessary information to guide and improve the prevention and control of AGE and FBD in Jamaica. Prevention of FBD is also very important to the sustainability of the largely tourism-dependent economy of Jamaica since traveller's diarrhoea (of which FBD is a common cause) and FBD have been reported among tourists to the Caribbean region, including Jamaica and have been shown to negatively affect the image and sustainability of the Caribbean tourism industry and their economies (14-16). Some episodes of diarrhoea are believed, however, to be caused by non-pathogenic factors, such as the change in cuisine as Caribbean meals are known to be much spicier than North American or European meals.

The monthly prevalence of AGE was estimated to be 4.03%, with a relatively equal distribution noted among both the genders. This value is at the lower end of the range of AGE prevalence previously reported by BOI studies conducted in other countries (10,17-21). Despite the lower prevalence, the burden of AGE on the population of Jamaica was demonstrated to be considerably underestimated based on the level of underreporting found for syndromic (64%) and laboratory-confirmed AGE (>99%) and the duration of illness and loss of productive days that resulted. The data confirm that there are many more cases of AGE occurring in the community than the current surveillance systems are detecting and that underreporting of both syndromic and laboratory-confirmed AGE to the national surveillance system was common. This finding implies that gaps and inefficiencies exist in the current surveillance system for AGE in Jamaica. Particular issues exist for laboratory-based AGE surveillance because of infrequent stool collection, difficulties with transportation of samples, inadequate specimen submission, and incomplete reporting of data to the surveillance system. There is, therefore, a need to improve the surveillance of AGE and considerations given to having an integrated surveillance system for AGE and FBD. The cultural phenomenon of the Jamaicans characterized by reluctance to provide a stool sample contributed to the low level of request made and less than 100% compliance with those requests, and this must be taken into account in assessing the performance of the laboratory surveillance functions. It is not expected that the surveillance system will detect 100% of the cases of AGE occurring in the communities in Jamaica but what this study has done is to provide us with an accurate underreporting multiplier, which will allow the calculation of more accurate data on prevalence and incidence and give a better appreciation of the magnitude of the problem. Further analysis of the additional factors collected in the population survey (not reported here) is required to understand the practices and risk and protective factors associated with AGE to facilitate policy formulation, programming, and interventions to address this issue.

**Table 5.** Healthcare-seeking behaviour among self-reported cases of acute gastroenteritis (AGE) in Jamaica, 2009

Healthcare-seeking behaviour/Use of medical treatment	Number of cases (%)
Sought medical/healthcare	
Yes	18 (36)
No	32 (64)
Type of healthcare facility visited (n=18)	
Private hospital	1 (5.6)
Public hospital	10 (55.6)
Private doctor's clinic	5 (27.8)
Health centre	3 (16.7)
Alternative health practitioner (e.g. Herbalist)	0 (0)
Traditional healer	1 (5.6)
Other	1 (5.6)
Hospitalized (n=18)	
Yes	2 (11.1)
No	16 (88.9)
Nights of hospitalization (n=2)	
Mean	3.5
Median	3.5
Medication prescribed (n=18)	
Yes	18 (100)
Type of prescription medication taken (n=18)	
Pain killer	6 (33.3)
Antibiotic	6 (33.3)
ORS	12 (66.7)
Other	4 (22.2)
Non-prescribed medication taken (n=50)	
Yes	17 (34)
No	30 (60)
Pain killer	4 (23.5)
Antibiotic	0 (0)
ORS	5 (29.4)
Bush medicines/home remedies (n=15)	5 (33.3)
Other	5 (29.4)
Cases with bloody diarrhoea (%)	5% (n=2)
Cases who sought medical care (%)	36
Stool sample requested from AGE case	3/18 (16.7%)
Cases submitting stool samples	2 (66.7%)

The highest monthly prevalence of AGE (14.6%) was among the 1-4 year(s) age-group, which is comparable with that in Cuba (21) and showed that the under-five children are at an increased risk for developing AGE (22-24). Age was the only sociodemographic variable that was found to be significantly associated with being a case of AGE. The corroboration of this finding in a Jamaican setting justifies the collation and monitoring of syndromic AGE data by age-groups (<5 years and ≥5 years) in routine syndromic surveillance. The higher prevalence and incidence of AGE in the younger age-group is not surprising as some of these children have recently been weaned from breastfeeding, which provided ideal immunological protection from diarrhoeal pathogens.

Only one-third (36%) of the self-reported AGE cases sought formal healthcare for their illness and, of them, only 11.1% (2/18) required hospitalization. While this trend may suggest that the severity of AGE in the Jamaican population is relatively mild, the frequency of healthcare-seeking behaviour for AGE in this study is still notably lower than in other studies and may also be reflective of cultural attitudes towards AGE and/or socioeconomic limitations to seeking healthcare. A higher proportion of persons (11%) in Jamaica required hospitalization compared to Cuba (<1%) (21) but the mean duration of hospitalization (3.5 nights) was comparable with that of Argentina (10). All persons who sought medical care required treatment, including oral rehydration therapy and/or antibiotics, and some who did not seek medical care self-treated their illness. Overall, a total of 68% of AGE cases sought treatment, which contributes to the overall cost of AGE as it can be a significant proportion of annual household income (25,26). Persons with AGE had symptoms for about 3.1 days, resulting in the loss of approximately 4 productive days, with 53.3% of cases requiring a caregiver for nearly 5 days. Extrapolating these data to the true incidence and prevalence of the disease will more accurately reflect the full economic impact of AGE. Therefore, despite the relatively mild presentation, it is likely that high costs are associated with AGE for the healthcare system, the individual, family, and the economy, including direct medical costs, loss of income and time spent by parents and guardians/caregivers looking after ill children (26-28). While an actual cost was not calculated yet for AGE at the time this paper was being written, the cost at all levels would contribute to a high economic burden from AGE and FBD (29,30).

Our data found that respiratory symptoms were frequently recorded as associated symptoms, which have been reported by other studies (30-31). Among all cases of AGE, respiratory symptoms (sneezing 38.6%, cough 37.2%) were the second and third most frequently-reported symptoms associated with the illness. One possibility explored by Hall *et al*. (31) is that viral illnesses often have similar non-specific clinical symptoms and, as such, diarrhoeal symptoms may be due to a respiratory illness opposed to an enteric cause. The opposite is also possible whereby respiratory symptoms experienced may be due to an enteric cause. Rotavirus, enteric adenovirus, and norovirus are common causes of diarrhoea and may be spread by both faecal-oral as well as respiratory routes. This should, therefore, be considered when analyzing the pathogen-specific aetiology to determine the estimated burden of specific FBD, particularly if majority of the burden from AGE was due to viral causes. It is also possible that a fair proportion of persons with AGE and respiratory symptoms may be experiencing mixed infections concurrently as is expected in children aged <5 years (32-34). This possibility has implications for the collation of syndromic data and sample collection in Jamaica as syndromic data are collated on both AGE and fever and respiratory symptoms. As such, surveillance data need to be validated periodically to ensure that persons who present with more than one syndrome are appropriately captured under all the relevant syndromes, and the relevant samples are taken for each syndrome.

The huge disparities between what is reported to the national surveillance system and the calculated estimates of the community burdens in this study clearly support the rationale for using the multiplier approach to quantify the level of underreporting of AGE (35-37). It also substantiates the need to integrate data from the various surveillance systems, i.e. syndromic and laboratory-based, to facilitate a more comprehensive picture of national disease trends. Interestingly, the multipliers and overall underreporting factor were found to be the largest when only the estimation of burden of AGE was calculated solely on laboratory-based data. The level of underreporting was more than 100 times greater than the underreporting factor when syndromic data were used as a single source and a little more than 6 times higher than the underreporting factor calculated when the combined data were utilized. This draws attention to weaknesses in the laboratory component of Jamaica's surveillance system. Less than one-fifth of persons who sought care were asked to submit their specimens. This is not that alarming as routine sampling for AGE is not required to be exhaustive (38), and the current recommendation to clinicians is that 10% of persons who attend should be sampled, similar to that practised in The Netherlands (39). Although the samples that had been submitted are of greater concern, three-quarters of those did not meet the criteria for the sample to be tested based on the testing protocol. Another issue is that, of all the positive samples isolated at the main laboratories, only 70% were reported to the Ministry of Health. These observations underline the fact that, although Jamaica has laboratory surveillance as part of their routine data-capture for AGE, it is not functioning as optimally as it could. To address these gaps, ongoing retraining for clinicians and laboratory personnel on the requirements for syndromic sample collection is needed. Additionally, a health information system that facilitates communication between the health facilities and the laboratories while maintaining the integrity and confidentiality of the data would also be greatly beneficial in streamlining the integration of the various arms of the surveillance system.

### Limitations

The authors recognize several limitations in this study. The overall response rate of 65.8% was lower than the expected 80%. Administrative challenges experienced in the implementation of the data collection, especially in the second phase, and difficulties with the questionnaire design could have contributed to the lower-than-expected response rate. This mainly resulted in incomplete questionnaire submitted. Additionally, amongst completed surveys, some questions were consistently omitted, which also contributed to the low response rate. However, population-based surveys typically have challenges with obtaining optimal response rates, and Jamaica's response rate is among the higher end in comparison with other recent BOI studies (17-21). In comparison with the national population data, the findings suggest an overrepresentation of females as a result of the low response rate—a similar result as that encountered in Argentina (10) and Canada (40).

### Conclusions

The level of underreporting found in this study indicates that the burden of AGE far exceeds our best assumptions based on routine data being collected. This clearly results in direct and indirect costs to patients and their families, the healthcare system as well as the wider society as loss of productivity has economic implications that impact all levels of society from the individual to the population. The high prevalence among the <5 years age-group—a vulnerable sub-population—indicate the need for health promotion interventions, including promotion of extended breastfeeding, to be targeted towards both prevention and provision of adequate care for this group and also enhancing education of parents/caregivers.

The burden of AGE has been validated from data presented in this research and opportunities for improvement identified. Jamaica's surveillance system already incorporates syndromic and laboratory data in the monitoring of AGE but efforts need to be made to strengthen this further, with particular attention given to strengthening laboratory surveillance. Further analysis of the pathogen-specific causes of foodborne illnesses, the risk factors, socioeconomic determinants, and routine practices associated with self-reported cases of AGE will be necessary to better guide the development of targeted interventions and policy-decisions towards improving the surveillance system to incorporate multiple sources of data and to apply (with ongoing review and updating) the underreporting multipliers for syndromic as well as laboratory components for the best available estimates. Other components of the surveillance system, e.g. hotel-based surveillance, need to be reviewed and strengthened in tandem with the syndromic and laboratory components.

## ACKNOWLEDGEMENTS

We acknowledge the following persons for their contributions to the development of the study proposal: Dr. Taraleen Malcolm, Dr. Nicola Skyers, and Ms Andriene Grant; Mr. Hubbert Sherrard from STATIN for assistance with the sample selection; Mr. Tenien Black for assistance with data-entry and cleaning; laboratory staff from NPHL, UHWI, and CRH for laboratory data; and PAHO/WHO Jamaica Office for support in obtaining the grant funds. We also thank the staff of the Regional Health Authorities for assistance with the population surveys. Special mention is made of the Community Health Aides, the Caribbean Epidemiology Centre (CAREC), the Public Health Agency of Canada, in particular Dr. Kate Thomas, and the Pan American Health Organization (PAHO), particularly Dr. Enrique Perez, for providing technical coordination for the study. We also acknowledge financial and material support from CAREC, PAHO/WHO, and IDRC.
